# Supplementary material for: Salicylate Sodium Suppresses Monocyte Chemoattractant Protein-1 Production by Directly Inhibiting Phosphodiesterase 3B in TNF-α-Stimulated Adipocytes
Source: Int J Mol Sci. 2022 Dec 24;24(1):320. doi: 10.3390/ijms24010320 (PMC9820166; doi:10.3390/ijms24010320)
Supplement: Supplementary file 1 [file ijms-24-00320-s001.zip › ijms-2082361-supplementary.pdf]

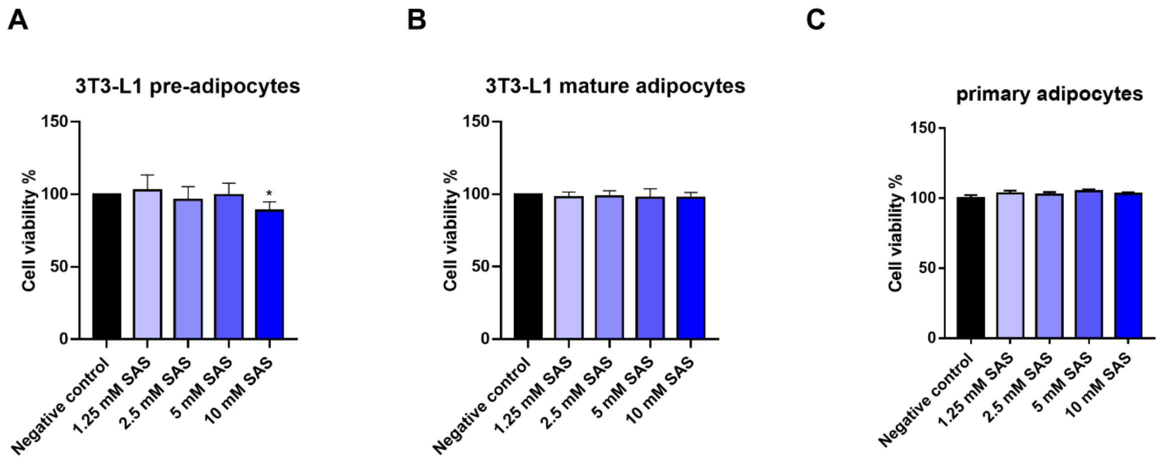

Figure S1. Effect of SAS on the viability of adipocytes. 3T3-L1 pre-adipocytes **(A)** or mature 3T3-L1 adipocytes **(B)** or primary adipocytes **(C)** were incubated with SAS at the indicated concentrations. Twenty-four hours later, the cell viability was evaluated by MTS assay. Data were shown as the mean ± SD (n = 3). $^{*}P < 0.05$ *vs.* normal control. SAS, salicylate sodium.

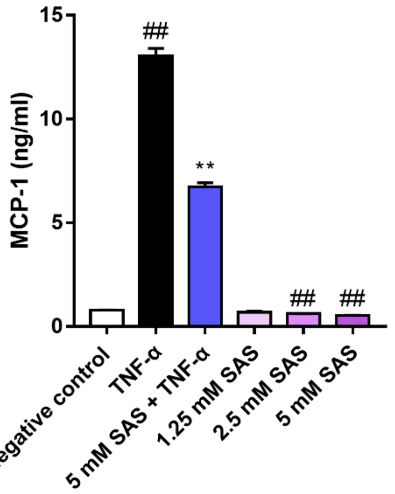

Figure S2. Effect of SAS on MCP-1 production of 3T3-L1 pre-adipocytes in the presence or absence of TNF-*α*. The cells were pretreated with SAS at different concentrations (0 - 5 mM) for 1 h, and then stimulated with mouse TNF-*α* (40 ng/ml) or culture medium at 37°C for 24 h. Supernatant MCP-1 was determined by ELISA. Data were shown as the mean ± SD (n = 3). ##$P < 0.01$ *vs.* negative control; **$P < 0.01$ *vs.* TNF-*α* alone. SAS, salicylate sodium.

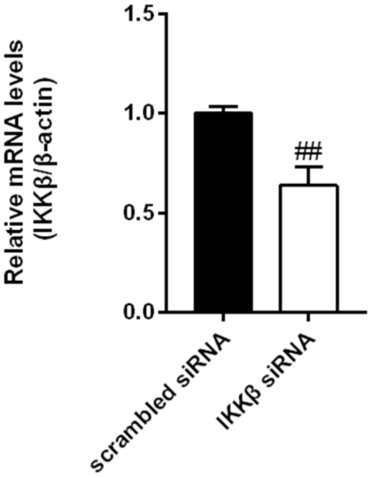

Figure S3. IKKβ mRNA levels in 3T3-L1 pre-adipocytes transfected with IKKβ siRNA or the scrambled siRNA. 3T3-L1 pre-adipocytes were cultured in 6-well plates (3×10^6 cells per well). Twenty-four hours later, the cells were transfected with IKKβ siRNA (3.33 μg/well), or the scrambled siRNA using transfection reagent EntransterTM-R4000 (5 μl/well) according to the manufacturer's protocol. Six hours later, the culture medium was changed to fresh medium, and the cells were cultured for further 48 h. The obtained target gene-deficient cells were confirmed by RT-qPCR. The primer sequences for IKKβ were as follows: 5′-GTGCCTGTGACAGCTTACCT-3′ (forward) and 5′-ACTGCGTTTGCACTTTTGCT-3′ (reverse). The primer sequences for β-actin were as follows: 5′-TGTTACCAACTGGGACGACA-3′ (forward) and 5′-AAGGAAGGCTGGAAAAGAGC-3′ (reverse). Data were shown as the mean ± SD (n = 3). $^{\#\#}P < 0.01$ *vs.* negative control.

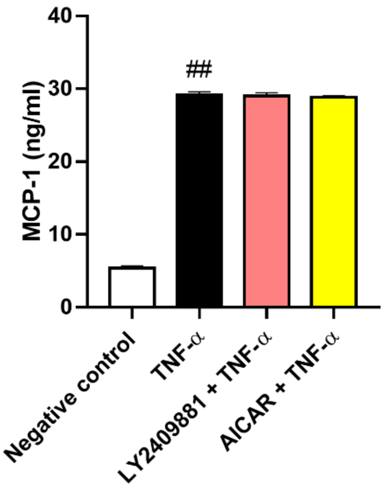

Figure S4. LY2409881 and AICAR cannot decrease MCP-1 release in TNF-α-stimulated 3T3-L1 pre-adipocytes. The cells were pretreated with LY2409811 (100 nM) or AICAR (100 μM) for 1 h and then stimulated with mouse TNF-α (40 ng/ml) at 37°C for 24 h. Supernatant MCP-1 was determined by ELISA. Data were shown as the mean ± SD (n = 3). $^{##}P < 0.01$ *vs.* negative control.

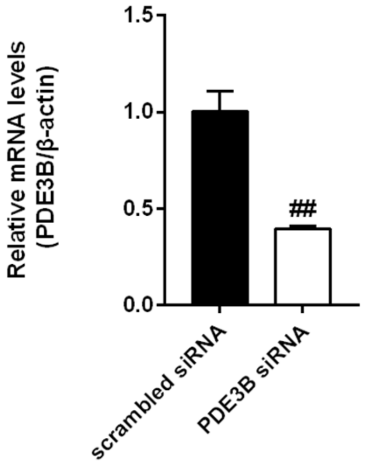

Figure S5. PDE3B mRNA levels in 3T3-L1 pre-adipocytes transfected with PDE3B siRNA or the scrambled siRNA. 3T3-L1 pre-adipocytes were cultured in 6-well plates (3×10^6 cells per well). Twenty-four hours later, the cells were transfected with PDE3B siRNA (3.33 μg/well), or the scrambled siRNA using transfection reagent EntransterTM-R4000 (5 μl/well) according to the manufacturer's protocol. Six hours later, the culture medium was changed to fresh medium, and the cells were cultured for further 48 h. The obtained target gene-deficient cells were confirmed by RT-qPCR. The primer sequences for PDE3B were as follows: 5′-GGTGATGGTGGTGAAGAA-3′ (forward) and 5′-AGTGAGGTGGTGCATTAG-3′ (reverse). The primer sequences for β-actin were as follows: 5′-TGTTACCAACTGGGACGACA-3′ (forward) and 5′-AAGGAAGGCTGGAAAAGAGC-3′ (reverse). Data were shown as the mean ± SD (n = 3). $^{\#\#}P < 0.01$ *vs.* negative control.

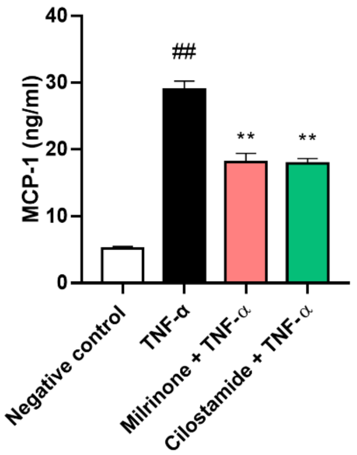

Figure S6. Milrinone and cilostamide can decrease MCP-1 release in TNF-*α*-stimulated 3T3-L1 pre-adipocytes. The cells were pretreated with 200 μM of milrinone or cilostamide for 1 h and then stimulated with mouse TNF-*α* (40 ng/ml) at 37°C for 24 h. Supernatant MCP-1 was determined by ELISA. Data were shown as the mean ± SD (n = 3). $^{##}P < 0.01$ vs. negative control; $^{**}P < 0.01$ vs. TNF-*α* alone.

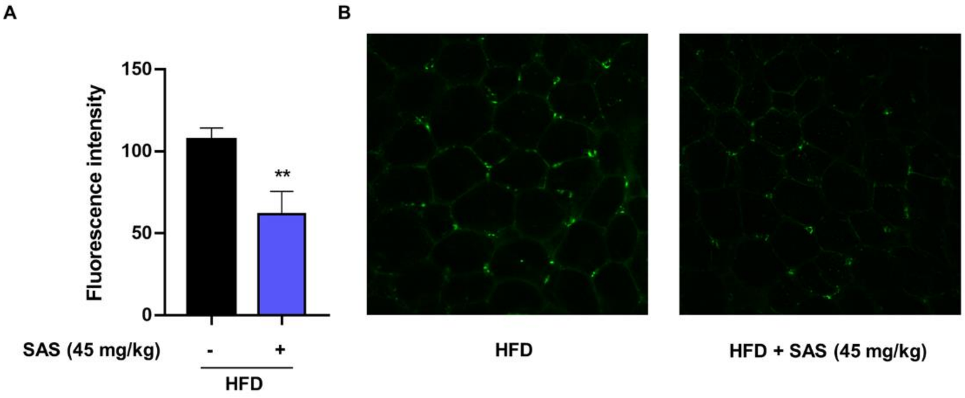

Figure S7. SAS impedes the recruitment of circulating monocytes into adipose tissue *in vivo* (n = 4). **(A)** The fluorescence intensity of monocytes in adipose tissue. Male C57BL/6N mice (18 g - 20 g) were fed with high-fat diet (HFD) for 18 weeks. The obtained obese mice (36 g - 40 g) with enough epididymal adipose tissues were subcutaneously injected with SAS (45 mg/kg/day) for 4 days. Circulating blood monocytes were labeled by intravenous injection of Fluoresbrite YG Carboxylate Microspheres. Three days later, the mice were euthanatized and adipose tissues around epididymis were collected, 1.5 g of adipose tissue was digested by collagenase I. The fluorescence value of digestive juice was read at λex 441 nm and λem 486 nm. $^{**}P < 0.01$ *vs*. HFD alone. **(B)** The photograph of labeled monocytes in adipose tissues. The sectioned adipose tissue (about 0.5 g) for photograph by a laser confocal microscopy using a 20 × objective. Fluorescence-labeled monocytes were shown in green. SAS, salicylate sodium.
